# Overexpression of the transporters At*ZIP1* and At*MTP1* in cassava changes zinc accumulation and partitioning

**DOI:** 10.3389/fpls.2015.00492

**Published:** 2015-07-09

**Authors:** Eliana Gaitán-Solís, Nigel J. Taylor, Dimuth Siritunga, William Stevens, Daniel P. Schachtman

**Affiliations:** ^1^Donald Danforth Plant Science Center, St Louis, MOUSA; ^2^University of Puerto Rico MayagüezMayagüez, PR, USA; ^3^University of Missouri Delta CenterPortageville, MO, USA; ^4^Department of Agronomy and Horticulture, University of Nebraska LincolnLincoln, NE, USA

**Keywords:** zinc, deficiency, plant, zinc transporters, cassava, storage root

## Abstract

Zinc deficiency in humans is a serious problem worldwide with an estimated one third of populations at risk for insufficient zinc in diet, which leads to impairment of cognitive abilities and immune system function. The goal of this research was to increase the bioavailable zinc in the edible portion of cassava roots to improve the overall zinc nutrition of populations that rely on cassava as a dietary staple. To increase zinc concentrations, two *Arabidopsis thaliana* genes coding for *ZIP1* and *MTP1* were overexpressed with a tuber-specific or constitutive promoter. Eighteen transgenic events from four constructs, out of a total of 73 events generated, showed significantly higher zinc concentrations in the edible portion of the storage root compared to the non-transgenic controls. The zinc content in the transgenic lines ranged from 4 to 73 mg/kg dry weight (DW) as compared to the non-transgenic control which contained 8 mg/kg. Striking changes in whole plant phenotype such as smaller plant size and chlorotic leaves were observed in transgenic lines that over accumulated zinc. In a confined field trial five transgenic events grown for 12 months showed a range of zinc concentrations from 18 to 217 mg/kg DW. Although the overexpression of zinc transporters was successful in increasing the zinc concentrations in 25% of the transgenic lines generated, it also resulted in a decrease in plant and tuber size and overall yield due to what appears to be zinc deficiency in the aerial parts of the plant.

## Introduction

Zinc is an essential mineral for both plants and animals. Zinc deficiency in humans is widespread, ranking fifth among the most important health risk factors in developing countries after obesity, iodine deficiency, iron deficiency, and vitamin A deficiency^1^ (WHO, 2002). Cassava (*Manihot esculenta* Crantz) is cultivated mainly for its edible starchy root and is an important calorie source for low-income populations in Sub-Saharan Africa and other tropical regions of the world. Cassava efficiently produces carbohydrates in greater quantities than either maize or sorghum under optimal growing conditions (El-Sharkawy et al., 1990), making it an attractive source of food. Although an excellent source of carbohydrates, cassava storage roots are low in protein, vitamins, and micronutrients such as zinc and iron. A survey of 600 cassava clones showed that the range of zinc concentration found in edible portions of the storage roots was between 2.6 and 37 mg/kg, with an average of 7.5 mg/kg (Chávez et al., 2005). To provide the minimum daily required amount of zinc for individuals eating between 500 and 1000 g of fresh cassava each day, it would be necessary to create a bio-fortified cassava product with at least six times higher zinc levels in the edible portion of the root (Sayre et al., 2011).

In plants, the first step in zinc uptake from the soil is uptake across the membrane of roots cells (Palmgren et al., 2008). Like other organisms, plants have multiple transporters that act in a regulated manner to control uptake, translocation, and storage of essential minerals. At least three different classes of transporters have been shown to be involved in zinc transport in higher plants. Those include a vacuolar zinc exchanger family named cation diffusion facilitators (CDF; van der Zaal et al., 1999; Drager et al., 2004; Kobae et al., 2004) that has six putative transmembrane domains, the plasma membrane ZIP transporters (ZRT, IRT-related proteins; Grotz et al., 1998) has eight putative transmembrane domains, and the heavy metal P-type ATPases or pumps (Hussain et al., 2004; Williams and Mills, 2005) that have eight putative transmembrane domains and are closely related to the proton pumping ATPases in the plasma membrane.

The first report of overexpression of a zinc transport molecule (van der Zaal et al., 1999) described constitutive expression of the *Arabidopsis MTP* gene (formerly called *ZAT*) which plays a role in zinc transport into vacuoles. *Arabidopsis* lines overexpressing this gene showed enhanced resistant to zinc levels up to 0.28 mM while controls showed chlorosis and slower development when exposed to these doses (van der Zaal et al., 1999). Transgenic plants also had increased zinc concentration in roots when exposed to high concentrations of this mineral (van der Zaal et al., 1999). Enhanced expression of *MTPs* due to increased gene copy number (Drager et al., 2004) is associated with hyper-accumulation of zinc in species such as *Arabidopsis halleri* that are capable of accumulating 100 times more zinc in their leaves than non-accumulating species such as *A. thaliana*. In contrast to *A. thaliana*, the hyper-accumulator *A. halleri* preferentially accumulates zinc in leaves rather than in the roots (Dahmani-Muller et al., 2000). The underlying control of where zinc is accumulated in *A. thaliana* and *A. halleri* is not fully understood.

Plasma membrane zinc transporters of the ZIP family have been identified in plants, animals, fungi, and bacteria (Gaither and Eide, 2001). The overexpression of zinc transport proteins has been shown to increase the zinc concentration in the roots of rice (Lee et al., 2010) and in seeds of barley (Ramesh et al., 2004; Tiong et al., 2014). The enhanced zinc uptake observed in the hyper-accumulating species *Thlaspi caerulescens* and *A. halleri has* also been correlated with increased *ZIP* gene expression (Pence et al., 2000; Becher et al., 2004).

Enhancing the bioavailable zinc content of cassava storage roots was a major goal of the BioCassava Plus project during the first phase (Sayre et al., 2011). Two approaches were considered for achieving the target of a sixfold increase in zinc concentration. One was to target overexpression of a protein that would bind to, and increase the content of bioavailable zinc within the storage root. Another was to modify uptake and transport of zinc by overexpressing zinc transporters. In contrast to knowledge concerning iron storage proteins such as ferritin, little is known about which proteins could be successfully overexpressed to bind to, and possibly increase total amounts of stored zinc. As described above, a more comprehensive picture of the zinc transporters was available when this project was initiated and therefore this led to the testing of transporter overexpression as part of this work.

In the present study, transgenic cassava plants overexpressing At*ZIP1* (plasma membrane zinc transporter) and At*MTP1* (a vacuolar membrane zinc transporter) under the control of the patatin promoter or the figwort mosaic virus (FMV) promoter were created and tested for altered zinc accumulation. Transgenic lines were characterized at the molecular level and grown in a growth chamber and under field conditions. Overexpression of these genes increased zinc concentrations in cassava starchy roots by 200–900%, which was also correlated with changes in zinc partitioning causing zinc deficiency in leaves. This report shows the feasibility of transporter overexpression and the need to use appropriate promoters for increasing the zinc concentration of edible plant parts. It also highlights the changes that occur in distribution of zinc in these transgenic lines and the future need to further optimize this approach to create high and high yielding crops.

## Materials and Methods

### Biological Material and Growth Conditions

*Manihot esculenta* cultivar 60444 was used for genetic transformation and in all experiments. Plantlets were cultivated on Murashige and Skoog basal medium (Murashige and Skoog, 1962), containing 20 g L^-1^ sucrose and 0.8% w/v Noble agar (MS2), in Petri dishes under controlled conditions at 28°C with 12 h light at 90 μmol m^-2^. After transformation, four to five plants per line were transferred to Fafard Mix 51 (Conrad Fafard, Inc., Agawam, MA, USA) in three inch pots as described by Taylor et al. (2012) and grown in a chamber at 65% humidity, 28°C with a 14 h photoperiod and 600 μmol m^-2^ light intensity. Growth chamber grown plants were fertilized twice per week using 15–17 Peat Lite, containing 200 ppm nitrogen and 0.04% chelated zinc. Soil-bed plants were grown for 8 months in a soil bed at Portageville, MO, USA. No fertilizer was applied to the soil-bed grown plants. Field testing of the transgenic lines was performed at the Isabela Agriculture Research station of the University of Puerto Rico Mayaguez in Northeastern Puerto Rico. *In vitro* plants were transferred to 4″ pots filled with Rain Forest potting mix soil and hardened for 2 months prior to planting in the field. The trial, with wild-type 60444 plants as control, was planted in a randomized block design with three reps and five plants/line/rep and was terminated after 12-months of growth. The distance between each plant was 1.5 m. At harvest, yield data comprising the number of storage roots, above ground and below ground mass, dry matter content and harvest index were measured. Also at harvest five storage roots/line/rep were washed, dried, and waxed on-site prior to shipping over-night to St. Louis for analysis while adhering to USDA APHIS regulations.

### Molecular Cloning, Construct Design, and Genetic Transformation

*Escherichia coli* strain DH-5α was used for plasmid manipulations and propagation of pGEM-Teasy (Promega, Madison, WI, USA). pCAMBIA2301 vector^2^ was modified by removal of the 35S promoter driven *uid*A expression cassette. At*MTP1* (At2g46800) and At*ZIP1* (At3g12750) genes were PCR-amplified from *A. thaliana*, cloned into pGEM-Teasy vector (Promega) and verified by sequencing. A total of five gene constructs were generated for integration into cassava. The PAT:At*MTP1* expression cassette in which the tuber specific class I patatin promoter from potato (Rocha-Sosa et al., 1989) was used to drive expression of At*MTP1*, and the PAT::At*ZIP1* expression cassette consisted of the patatin promoter driving At*ZIP1*(At3g12750). In addition, the FMV promoter was fused to At*MTP1* and At*ZIP1* to generate FMV:At*ZIP1* and FMV:At*MTP1*, respectively. A final construct was produced carrying both zinc genes within the same region of the T-DNA to make PAT:At*ZIP1*- PAT:At*MTP1*.

All pCAMBIA2301-based transformation vectors were mobilized into *Agrobacterium tumefaciens* strain LBA4404 by electroporation and used for delivery of T-DNA into plant cells. Friable embryogenic callus (FEC) of cultivar 60444 was produced and used for the production and recovery of transgenic plants as described by Taylor et al. (2012).

### Molecular Analysis of Transgenic Lines

Leaves from tissue culture plantlets were used as starting material for DNA extraction (Dellaporta et al., 1983). 25–50 ng of DNA was used for gene specific PCR to amplify At*ZIP1* (F; 5′-TTCTAGAATGTCTGAATGTGGATGTTT-3′, R: 5′- AGGTNACCTCAGGCCCAGATGGCGAGGA-3′) and At*MTP1* genes (F: 5′-AGGATCCATGGAGTCTTCAAGTCCCCA-3′, R: 5′-TGGTNACCTTAGCGCTCGATTTGTATCG-3′). Amplification conditions to detect At*ZIP1* and At*MTP1* genes in the transgenic plants were 94°C 1 min, 58°C 1 min, 72°C 1.5 min) for 30 cycles followed by a final incubation at 72°C for 10 min.

Starchy tuberous roots, leaves, and fibrous roots were analyzed for expression of the At*ZIP1*, and At*MTP1* transgenes in transformed plant lines. The peel layer was separated from the storage parenchyma tissue and analyzed separately. For expression analysis, tissue was freeze-dried and total RNA extracted from 300 to 500 μg of lyophilized material as per Ernst et al. (2010). Pellets containing RNA were washed twice with 1 mL of ice cold 80% EtOH, vortexed and centrifuged for 5 min at 16,000 rcf 4°C. RNA was resuspended in 40 μL of 0.1 mM Tris-EDTA. When needed, a DNAse was added to total RNA using Promega DNAse following manufacturer instructions. After precipitation, RNA was resuspended in DEPC water and 10 μg of total RNA loaded onto formaldehyde/MOPS 1.2% (w/v) agarose formaldehyde gel. Transfer of nucleic acids was performed as described by Maniatis et al. (1982) on a Hybond-N^+^ membrane (Amersham Life Science, USA). cDNA fragments of the *AtMTP1* (1,200 bp) and *AtZIP1* (1,077 pb) genes were labeled with [^32^P]alpha dCTP by random primer and used as probes for Northern blot hybridization.

T-DNA copy number of transgenic over-accumulating plant lines was determined by Southern blot analysis. DNA from leaves was extracted following the Dellaporta method (Dellaporta et al., 1983) using two and half grams of ground fresh leaf tissue as starting material. The pellet was dissolved in Tris-EDTA (10:1) containing 10 μg/mL of RNAse. RNA-free DNA was extracted twice with Phenol:Chloroform (24:1), followed by two washes of chloroform and finally disolved in 250 μL of TE (10 mMTris, 1 mM EDTA). Twenty micrograms of HindIII- or BamHI-digested DNA was separated on a 0.9% agarose gel, blotted on nylon membrane (Hybond-N^+^, Amersham Life Science), and probed with [^32^P] alpha dCTP labeled coding regions as described above.

### Analysis of Plant Tissue for Accumulation of Zinc

Levels of zinc within plant tissues were determined from plants grown for 4 months in the growth chamber, from 8 months-old plants grown in a soil bed under greenhouse conditions at University of Missouri, Portageville, MO, USA and from 12 months-old plants grown under confined field trial conditions at University of Puerto Rico Mayaguez, Mayaguez, PR, USA. No fertilizer was applied to the soil-bed or field-grown plants. Three plants per transgenic event were analyzed for zinc concentrations within leaves, fibrous roots, and storage roots. Soil was carefully removed from fibrous and storage roots to avoid zinc contamination. Storage roots were peeled to separate the fleshy parenchyma from the peel layer. The concentration of zinc in leaves was determined from tissue of the youngest fully expanded leaf (YFEL), normally positioned as the fourth to fifth leaf below the apical meristem. All tissues were washed in deionized water and Milli-Q water and oven-dried at 60°C for 1 week prior to analysis. Dried material was chopped, weighed and digested in nitric acid by adding 7 mL of nitric acid and heating for 3 h at 98°C in a hot block (Environmental Express, Pleasant, SC, USA). After digestion, final volumes were measured and zinc concentrations determined by Atomic Absorption Spectrometry (AAnalyst 300, Perkin Elmer, Waltham, MA, USA). The concentration of iron was measured by inductively coupled plasma-mass spectrometry (ICP-MS). Analysis of variance (ANOVA) and Spearman Correlation were performed using GraphPad Prism 6 (GraphPad software Inc., San Diego, CA, USA).

### Time Course for Zinc Distribution in Cassava Plantlets

The distribution of zinc was determined by tracing the movement of ^65^Zn (Medical Department, Radionuclide, and Radiopharmaceutical Research Division, Upton, NY, USA) in cassava plantlets using a scintillation counter. Plantlets were grown in 50 mL plastic tubes (Falcon) with 10 mL of MS2 medium solidified with 2 g/L Phytogel for 4 weeks, after which time they were carefully removed and the roots rinsed three times in Milli-Q water. Plantlets were moved to a clean 50 mL tube containing 3 mL of aerated MS2 liquid medium supplemented with ^65^Zn (62 KBq) at a total zinc concentration of 30 μM ZnSO_4_ for 24 h (labeling phase). After the labeling phase, roots were washed three times in Milli-Q water, desorbed for 15 min in CaCl_2_ and washed a further three times in Milli-Q water. Labeled plantlets were transferred to 50 ml of non-radioactive liquid medium with continued aeration. Zinc uptake and translocation was determined at 24 and 48 h after completion of the labeling phase. Three plants per transgenic line were analyzed at each time point. Each plant was divided into leaves, roots, stem, and smallest leaf (tip leaf); fresh weigh measured for each section and tissue was immersed in scintillation liquid for quantification. ^65^Zn present in plant tissues was quantified using a liquid scintillation counter LS 6000TA (Beckman Coulter, Indianapolis, IN, USA).

## Results

### Production of Transgenic High-Zinc Cassava Plants

*Agrobacterium*-mediated genetic transformation (Taylor et al., 2012) with five gene constructs containing *AtZIP1* and *AtMTP1* genes resulted in a total of 217 putative independent transgenic plant lines recovered from tissues selected on antibiotic-containing medium. Sixty putative transgenic events were recovered carrying the FMV:At*ZIP1* construct, 34 from FMV:At*MTP1*, 46 from PAT:At*ZIP1*, 38 from PAT:At*MTP1*, and 39 from FMV:At*ZIP1*-PAT:At*MTP1*. PCR analysis was performed on all these lines and confirmed that greater than 86% of the putative transgenic were positive for presence of the transgenes (data not shown). PCR-positive plant lines were planted to soil in pots for establishment in the growth chamber. Of the 217 plants regenerated, 47% of PAT:At*MTP1*, 44% of PAT:At*ZIP1*, 68% of FMV:At*ZIP1*, 22% of FMV:At*MTP1*, and 10% of FMV:At*ZIP1*-PAT:At*MTP1* plant lines survived and formed starchy roots whereas the remainder died in the greenhouse or did not form tubers within 4 months after transfer to soil.

### Zinc Analysis in Tissues of Cassava Transgenic Events Grown in Growth Chambers

Zinc concentrations of four different tissues were determined from transgenic plants that developed starchy roots after 4 months in the growth chamber. Twenty-one events of PAT:At*ZIP1*, 18 events of PAT:At*MTP1*, 13 FMV:At*ZIP1* events, 23 FMV:At*MTP1*, and four FMV:At*ZIP1*-PAT:At*MTP1* transgenic events were analyzed for zinc concentration in leaves, fibrous roots and the starchy storage parenchyma and peel layer of the storage roots. **Table 1** summarizes the range of zinc concentrations measured in the four tissue types in different transgenic lines for the five gene constructs as well as the non-transgenic control. All numbers in **Table 1** represent a mean of three individual plants. The Supplementary Table S1 contains the means and variation for each event from each line.

**Table 1:** Zinc concentrations in plant tissues from plants grown in grow chambers.

Genetic background	Storage parenchyma (mg/kg, DW)	Peel (mg/kg, DW)	Fibrous roots (mg/kg, DW)	Leaves (mg/kg, DW)	#Tuberized events analyzed
Wild type^1^	6.3–9.5	15.9–58.2	54.3–127.8	21.3–50.9	Multiple wild type plants
FMV:At*MTP1*^2^	3.9–24.4	6.8–67.0	59.7–170	9.3–59.4	23
PAT:At*MTP1*^2^	3.8 – 34.1	6.2–72.0	66.1–218.7	7.7–44.3	18
FMV:At*ZIP1*^2^	5.1–73.3	7.9–83.5	51.1–463.0	9.1–40.0	13
PAT:At*ZIP1*^2^	4.1–45.1	8.4–62.3	52.0–297.0	10.5–46.6	21
FMV:At*ZIP1/*PAT:At*MTP1*^2^	7.2–9.0	11.1–87.7	77.7–174.9	9.3–44.2	4

*The range of mean values (*n* = 3 for each mean; min to max) of zinc concentration from different events are shown. The means represent an average from three plants analyzed per event. Wild type control plants of cultivar 60444 were grown together with every set of transgenic plants under the same conditions. DW denotes Dry weight*.

*^1^Wild type controls of the cultivar 60444 were grown together with every set of transgenic lines tested in the greenhouse. Each number in the range of values represents the mean of three plants harvested*.

*^2^Each value in the transgenic events represents the mean of three plants per event for each line*.

**FIGURE 1:**
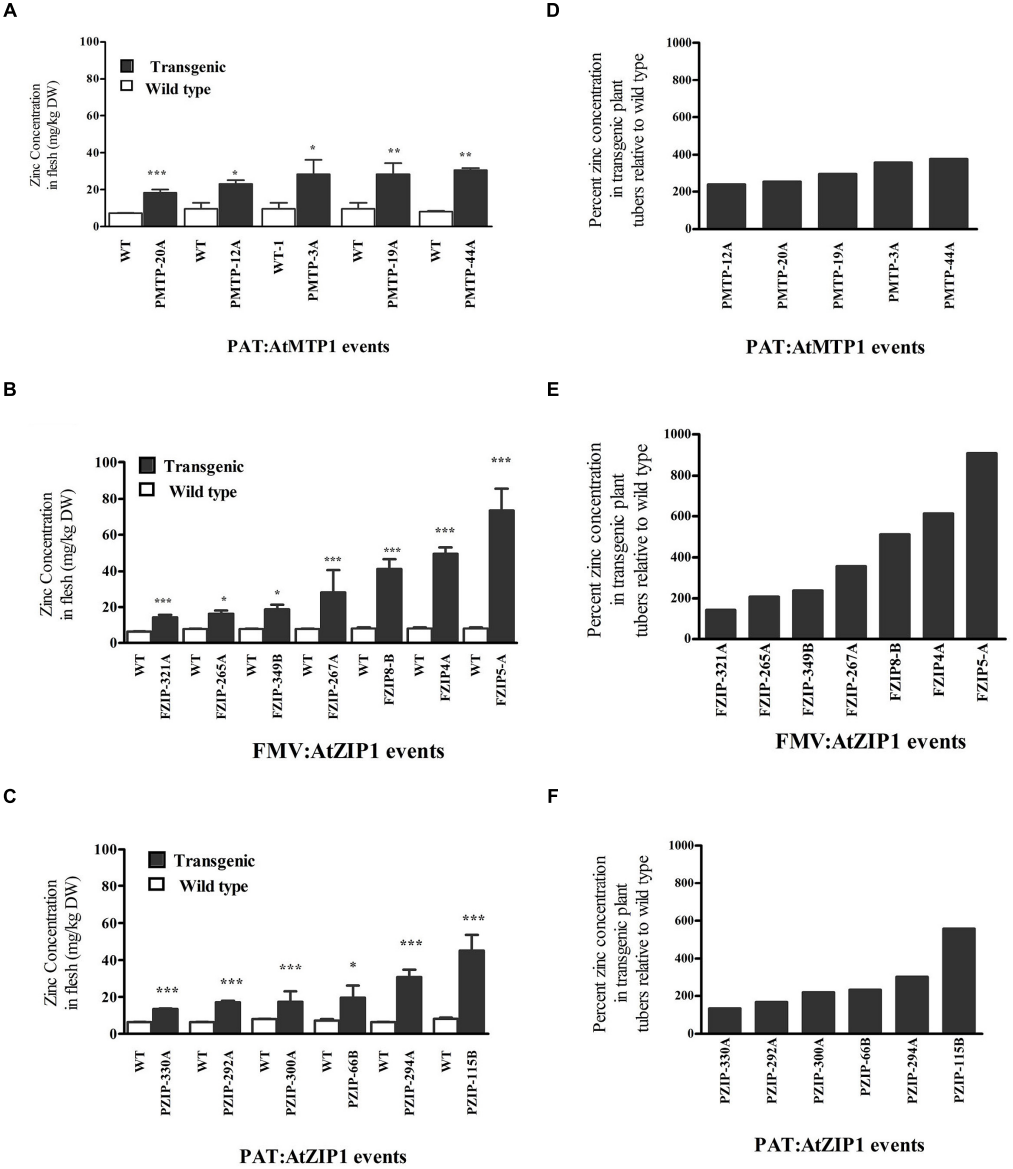
**Zinc concentration **(A–C)** and percentage increase in zinc concentration with respect to wild type **(D–F)** in 4-months-old cassava transgenic storage roots from growth chamber grown plants.** Values are mean zinc content normalized for dry weight (DW) of three independent samples, error bars indicate SD. Comparison is between wild type and transgenic events and indicate the different levels of statistical signficance ^∗^*P* < 0.05, ^∗∗^*P* < 0.01, ^∗∗∗^*P* < 0.001. FZIP denotes FMV:ZIP1 lines, PZIP denotes PAT:ZIP1 lines.

Eighteen lines consisting of six PAT:At*ZIP1*, five PAT:At*MTP1*, and seven FMV:At*ZIP1* (**Figure 1**), showed significantly higher zinc concentrations in their storage root parenchyma tissue compared to the non-transgenic controls (**Figures 1A–C**). None of the transgenic events of FMV:At*MTP1* showed a statistically significant increase in zinc concentration in the storage root as compared to controls (**Table 1**). Both transgenes were capable of driving increased accumulation of Zn in storage parenchyma but promoter choices were critical for the *AtMTP1*. *AtZIP1* under control of the patatin promoter resulted in higher maximum zinc levels, reaching almost six hundred percent (**Figure 1F**) higher than the controls, compared to only a 400% increase in plants expressing *AtMTP1* under the control of the same promoter (**Figure 1E**). The highest zinc accumulation was detected in storage roots of events overexpressing *AtZIP1* under control of the constitutive FMV promoter, in which concentrations reached 75 ppm, an 800–900% increase compared to the non-transgenic controls (**Figures 1B,E**). No significant increase in zinc accumulation was detected in any of the four transgenic plants containing the two gene construct FMV:At*ZIP1*- PAT:At*MTP1* (**Table 1**).

Transgenic plants were also analyzed for zinc concentration in their leaves, fibrous roots, and peel (**Table 1**). Zinc levels were found to be elevated in fibrous roots of transgenic plants with either *AtZIP1* or *AtMTP1* compared to controls. The largest change in zinc concentrations in these tissues was approximately 3.5 times greater in plants expressing FMV:At*ZIP1*. Only moderate accumulation was detected in the storage root peel layer, while levels in leaf tissues were reduced by as much as 50% compared to the non-transgenic controls (**Table 1**). Spearman correlation analysis of zinc concentrations between fibrous roots and leaves for all transgenic events was significantly negative (*r* = -0.63, ^∗∗∗^*p* < 0.0001). The zinc concentration of storage roots and peel showed a positive significant correlation across all transgenic events and in the subset of over-accumulating events (*r* = 0.4, ^∗∗∗^*p* < 0.0001, and *r* = 0.56^∗^, *p* = 0.016, respectively). Correlations between zinc concentrations in storage roots and fibrous roots and between storage roots and leaves were not significant across the complete data set analyzed.

### Transgene Integration and Expression of *AtMTP1* and *AtZIP1* in Cassava Tuberous Roots

**FIGURE 2:**
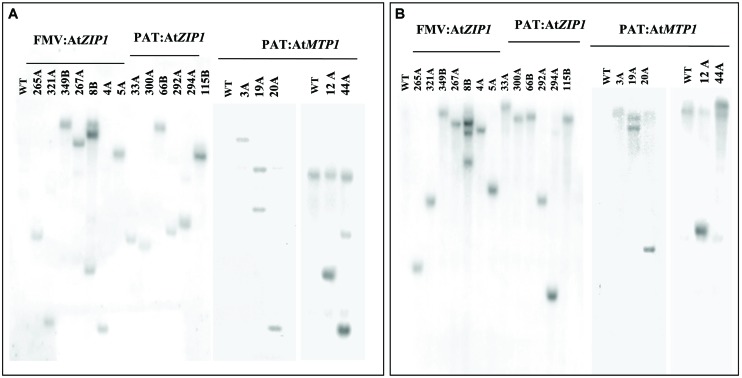
**Copy number determination using Southern blot hybridization of genomic DNA samples isolated from leaves of wild type and transgenic lines disgested with HindIII **(A)** and BamHI **(B)**.** WT, wild type.

**FIGURE 3:**
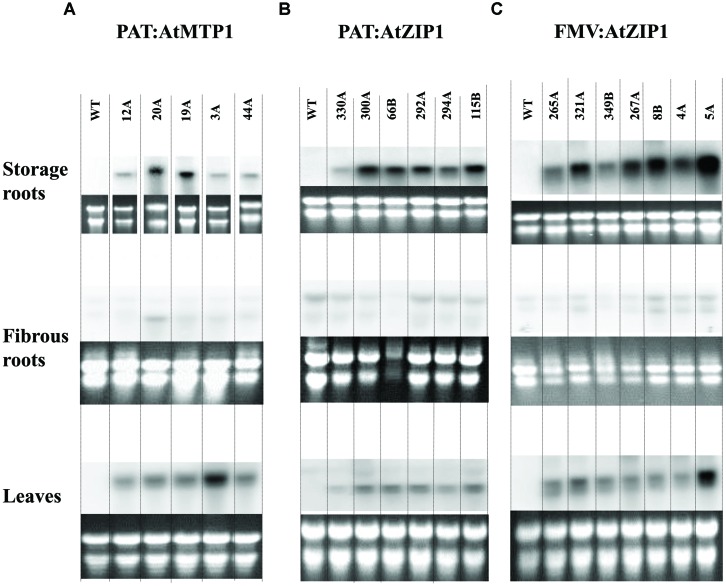
**Northern blot analysis for transgenic expression of *AtMTP1* and *AtZIP1* in storage parenchyma or tuberous roots, fibrous roots and leaves of 18 transgenic events.** Transcript levels of the At*MTP1* and At*ZIP1* driven by patatin promoter **(A,B)** and FMV promoter **(C)** in 4-month-old plants grown in a growth chamber. A 10 μg aliquot of total RNA was used for Northern analysis. WT, wild type control and multiple transgenic events for each construct.

Confirmation of transgene integration was confirmed by Southern blot analysis. Transgenic lines mostly had one to two copies of integrated T-DNA with only one line showing integration of three copies (**Figure 2**). Northern blot analysis was performed on all 18 Zn accumulating plant lines shown in **Figure 3** to confirm expression of At*MTP1* and At*ZIP1* transcripts in different plant parts. RNA from 4-months-old plants was loaded across the blot from lines containing high zinc concentrations to those containing lower concentrations in the starchy roots (**Figure 3**) to visualize any relationship between zinc concentration and transgenic RNA expression. All zinc accumulating events showed strong transgene expression (**Figure 3C**, upper panel). Levels of expression were similar for transgenic plants containing cassettes driven by the patatin and FMV promoters, with strongest expression seen in the storage root tissue, followed by the leaves and then fibrous roots. Data indicate that the FMV promoter was driving expression of *AtZIP1* to higher levels than the patatin promoter. This correlates with the higher levels of zinc accumulation seen in storage roots for FMV:*AtZIP1* events compared to PAT:*AtZIP1* (**Figure 1**). While transgene expression across all tissue types is expected in the case of the constitutive FMV promoter, the analysis of RNA transcripts showed that the patatin promoter is effective for driving transgene expression in the storage roots but expression was not restricted to this organ in plants 4 months after transfer to soil in the greenhouse.

### Analysis of Soil Bed and Field Grown Zinc Transporter Overexpressing Transgenic Events

**FIGURE 4:**
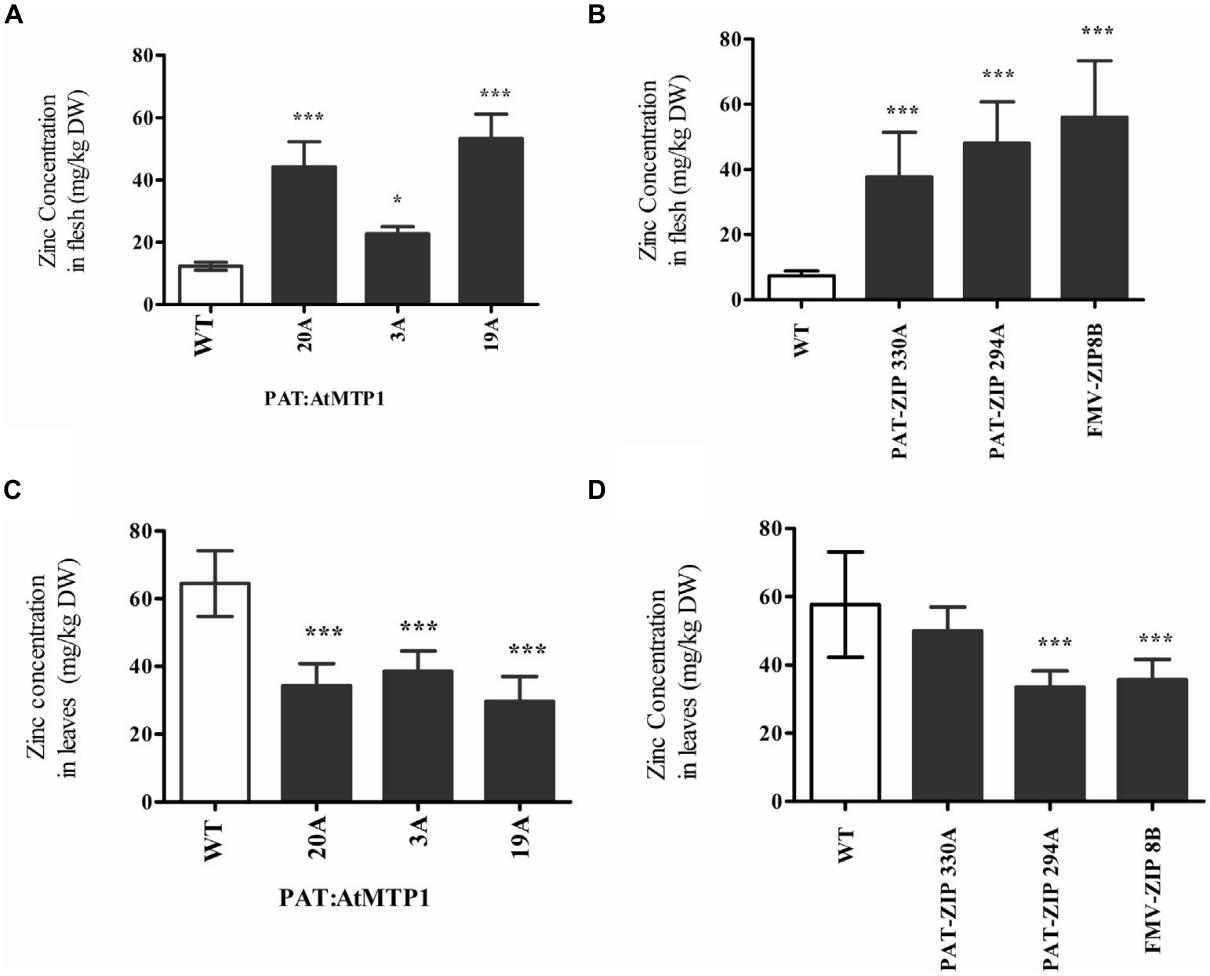
**Zinc concentration in the edible part of the storage roots **(A,B)** and leaves **(C,D)** of six independent transgenic PAT:At*MTP1*, PAT:At*ZIP1*, and FMV:At*ZIP1* events after 6–8 months of growth in a greenhouse soil bed.** Data represent the mean zinc content normalized for DW of seven independent samples, error bars indicate SD. Comparison is between wild type and transgenic events and indicate the different levels of statistical significance ^∗^*P* < 0.05, ^∗∗∗^*P* < 0.001.

In order to produce more mature plants with larger storage roots, transgenic lines from three constructs were established and grown in soil beds within a greenhouse at the University of Missouri, Portageville, MO, USA. Zinc levels in leaves and tuberous storage roots were assessed after 8 months of growth in the 50% of plants that formed tubers which included three zinc over accumulating transgenic lines from construct PAT:At*MTP1*, two from PAT:At*ZIP1* and one from FMV:At*ZIP1*. Soil bed grown plants showed significantly higher zinc concentration than the wild type control in their storage roots, with mean values ranging from 22 to 53 mg of zinc per kilogram dry weight (DW; **Figures 4A,B**). These zinc concentrations were more than twice that obtained from plants grown in pots in the growth chambers (**Figure 1A**). Zinc concentrations in leaves were significantly lower than the control for all three *AtMTP1* expressing lines and in PAT:At*ZIP1* line 294A and FMV:At*ZIP1* line 8B (**Figures 4A,B**). These transgenic plant lines showed an approximate 50% reduction in leaf Zn levels compared to non-transgenic controls at 28–38 mg/kg DW zinc (**Figures 4C,D**).

**FIGURE 5:**
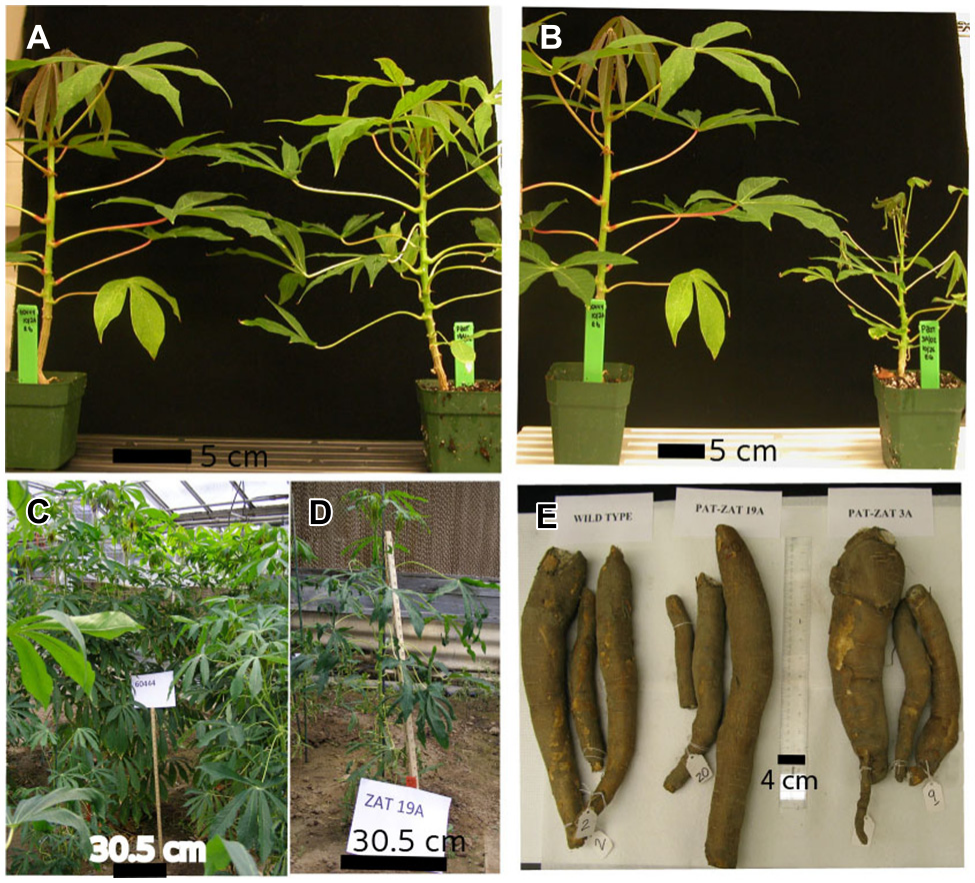
**Above ground plant phenotype in the green house **(A,B)**, soil bed (**C** – Wild type, **D** – transgenic PAT:At*MTP1* line).** Starchy tubers at 12 months after planting in soil bed trials in Portageville **(E)**.

Changes in phenotype were observed in transgenic plant lines that over-accumulated more than twice the storage root zinc concentration found in the non-transgenic controls. The changes in phenotype included smaller plant size and chlorotic leaves. This phenotype was observed in plants grown in the growth chamber (**Figures 5A,B**) and also in plants grown in soil beds (**Figures 5C,D**) where a stunted phenotype was also observed.

**FIGURE 6:**
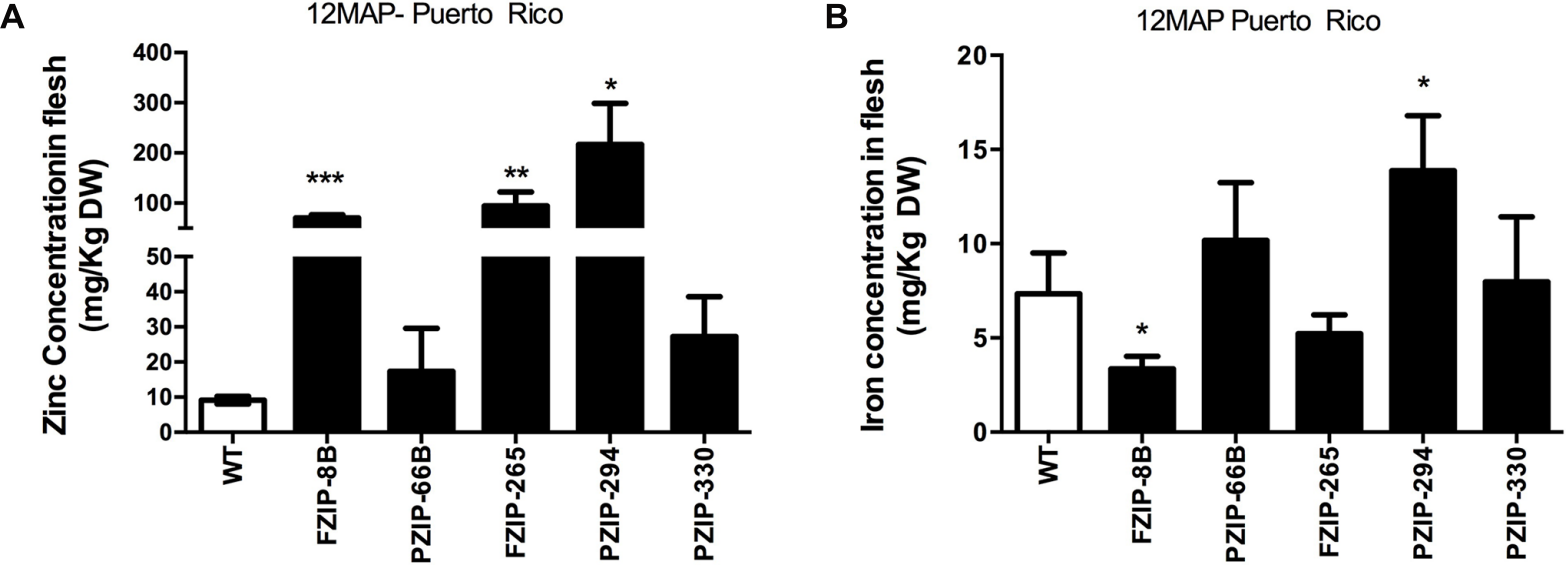
**(A)** Zinc and **(B)** iron concentration in flesh of five independent transgenic PAT:At*ZIP1* (PZIP) and FMV:At*ZIP1* (FZIP) events after 12 months of growth in fields at Puerto Rico. Data represent the mean zinc content of three replicated plots, error bars indicate SD. Unpaired *t*-test comparison wild type vs. transgenic event. ^∗^*P* < 0.05, ^∗∗^*P* < 0.01, ^∗∗∗^*P* < 0.001. MAP, months after planting. FZIP denotes FMV:ZIP1 lines, PZIP denotes PAT:ZIP1 lines.

Zinc accumulation in a set of transgenic events expressing the *AtZIP1* transgene under control of the patatin and FMV promoters was also assessed in a confined field trial at The University of Puerto Rico Mayaguez. Five transgenic events were grown for 12 months and zinc and iron concentrations determined in tuberous roots (**Figures 6A,B**). Analysis showed a range of zinc concentrations in the transgenic events from 17.5 to 217 mg/kg DW (**Figure 6A**) and 3.4–14 mg/kg DW for iron concentration (**Figure 6B**). Zinc in the transgenic storage roots were higher than the wild type control with some significantly greater than the wild type (*p* < 0.05 *t*-test). Non-transgenic plants at 12 month after planting had a mean height of 270 ± 17 cm while transgenics ranged from 94 to 198 cm (SD ± 15 to ± 42) depending on the individual. Likewise, storage root yield at harvest time indicated a yield penalty associated with the higher storage root zinc accumulation, such that the non-transgenic controls yielded an average of 6.95 ± 2.10 Kg/plant (*n* = 8, ± SD), the transgenic plants produced yields between 0.24 ± 0.10 and 1.62 ± 1.03 Kg/plant (*n* = 8, ± SD).

### Determination of Zinc Partitioning in Transgenic Plants Using ^65^Zn

Radiolabelled zinc (^65^Zn) was utilized to study uptake and partitioning in transgenic plants and the wild type control. Zinc partitioning, was expressed as a partition quotient (PQ) value as used by Waters and Grusak (2008) to represent the proportional mineral content in a tissue relative to the proportional DW of that tissue. In the present work a variation of the original PQ value was used in which the proportional radionuclide content in a tissue was obtained to allow the comparison of ^65^Zn distribution between the plants independent of their size. Plantlets derived directly from tissue culture medium using two lines per each of the constructs FMV:At*ZIP1*, PAT:At*ZIP1*, FMV:At*MTP1*, and PAT:At*MTP1* were analyzed at 24 h (T0) and 48 h (T2) after incubation with ^65^Zn (**Table 2**). No statistically significant difference was seen for the zinc PQ between fibrous roots of the wild type and transgenic lines at T0 (24 h after exposure) and at T2 (48 h after exposure) with the exception of transgenic plants from F*ZIP1*15 line which showed a significant increase in the amount of zinc localized in the roots with respect to the wild type (PQ = 265.7, **Table 2**). Zinc mobilization and accumulation in the lower stem (**Table 2**) was significantly reduced in all transgenic plants compared with the wild type at 24 h after exposure. Only line PZIP-300 did not show significantly lower PQ values at 48 h after exposure. Top leaf PQ values were mostly significantly lower at 48 h after exposure, with five out of eight lines showing such differences when compared with the wild type control.

**Table 2:** ^**65**^Zn uptake in cassava transgenic lines.

Partition Quotient (PQ)					
	**Root**	**Lower stem**	**Upper stem**	**Upper leaves**	**Top leaf**

**Time 0 (24 h exposure)**
Wild type	250.9 ± 48.9	247.6 ± 68.8	52.9 ± 20.8	10.9 ± 5.4	82.5 ± 13.7
FZIP 8	277.1 ± 19.3	118.4 ± 13.8^∗∗^	32.3 ± 14.4	9.9 ± 1.8	16.9 ± 5.5^∗∗∗^
FZIP 321	308.7 ± 23.8	82.7 ± 28.3^∗∗∗^	42 ± 17.6	19.7 ± 4	78.7 ± 5.9
FZIP 115	259 ± 18.2	79.3 ± 10.3^∗∗∗^	48 ± 23.1	17.7 ± 8.6	50.7 ± 5.7
PZIP 300	301.7 ± 40.4	139 ± 23.6^∗^	42.7 ± 7	16 ± 11.3	95.3 ± 15.3
FMTP 15	190.3 ± 20.3	93.7 ± 18^∗∗∗^	34.7 ± 4.7	63 ± 2.6^∗∗∗^	108.7 ± 18.2
FMTP 38	233.3 ± 21.8	88 ± 16.6^∗∗∗^	58.7 ± 18.1	34.3 ± 5.7^∗^	62.7 ± 9.3
PMTP 3A	196 ± 9.5	85 ± 18.4^∗∗∗^	45 ± 17.3	45.7 ± 11.7^∗∗∗^	39 ± 8.2^∗∗^
PMTP 20A	257.7 ± 26.8	146.3 ± 27.1^∗^	27.3 ± 8.4	23 ± 4.4	53.7 ± 10.1
**Time 2 (48 h exposure)**
Wild type	122.5 ± 19.7	232.3 ± 37.1	78.4 ± 11.6	59.2 ± 4.7	150 ± 20.8
FZIP 8	204.7 ± 31.6^∗^	133.8 ± 15.3^∗∗^	47.4 ± 6.5^∗^	23.8 ± 11.7	70.7 ± 5.3^∗∗∗^
FZIP 321	197 ± 37.6	64.3 ± 3.1^∗∗∗^	51.7 ± 5.5	74.3 ± 10.2	108 ± 11.5^∗^
FZIP 115	265.7 ± 44.8^∗^	78.7 ± 12.4^∗∗∗^	56 ± 11.3	24 ± 10.5	28 ± 3.6^∗∗∗^
PZIP 300	212.7 ± 51.4	171.3 ± 24.8	71 ± 2	40.7 ± 12.6	164.3 ± 17.6
FMTP 15	145.7 ± 7.2	147.3 ± 13.6^∗∗^	62.3 ± 5.7	60.3 ± 11.8	131 ± 11.8
FMTP 38	166.7 ± 11.7^∗^	95 ± 3.5^∗∗∗^	78.3 ± 8.5	61.3 ± 2.1	74 ± 11.1^∗∗∗^
PMTP 3A	169 ± 51.1	106 ± 31.2^∗∗∗^	68.7 ± 21.5	53 ± 24.2	82.3 ± 4.9^∗∗∗^
PMTP 20A	176 ± 17.8^∗^	125.3 ± 18.6^∗∗∗^	45 ± 1^∗^	58.7 ± 12	116 ± 17.1

*Plants were collected at different time points (after 24 h of exposure, T0 and after 48 h of exposure, T2). Plantlets from tissue culture were dissected into: labeled roots; half lower stem portion (Lower S), half upper stem portion (Upper S), leaves in the half upper stem portion (Upper L) and the smallest leaf at the top (Top leaf). Data shown as the mean of PQ (% of cpm uptake per tissue relative to the total divided by % mass per tissue relative to the total weight) of three plantlets per event and the SD. Statistical comparison is between wild type and transgenic events ^∗^*P* < 0.05, ^∗∗^*P* < 0.01, ^∗∗∗^*P* < 0.001. FZIP: FMV:ZIP1 lines, FMTP: FMV:MTP1 lines, PZIP: PAT:ZIP1 lines, PMTP: PAT:MTP1 lines*.

## Discussion

In most developing countries, plant-derived foods are the major sources of minerals for the majority of the population. Concentrations of iron, zinc, and iodine are low in plant compared to animal derived foods (Waters and Sankaran, 2011), leading to more than two billion people suffering from or at risk of micronutrient malnutrition^3^ (WHO/WFP/UNICEF, 2007). Few reports are available on the mineral concentrations in different parts of cassava which are likely to vary depending on growing conditions. In a comprehensive study, Chávez et al. (2005) reported mineral concentrations in 600 genotypes, showing zinc to be present at an average of 7.5 mg/kg DW in the storage roots which is the edible part of the plant.

Increasing the mineral content of crops requires either increased uptake from the soil or changes in the way minerals are partitioned in the plant. A crop breeding solution to elevated mineral content is a very a long-term process, especially in a heterozygous out-crossing species such as cassava that is usually clonally propagated and depends mainly of the mineral pools available in soil and the genetic variability present in the crop (Zhu et al., 2007; Cakmak, 2008). An alternative strategy is the use of transgenic approaches to enhance levels of zinc and other micronutrients within edible plant parts (Sayre et al., 2011). In the work presented here, increased concentrations of zinc were achieved in the storage roots of cassava by expressing the zinc transporter *ZIP1* (Grotz et al., 1998) and the vacuolar zinc transporter *MTP1* (van der Zaal et al., 1999) genes from *A. thaliana*. Although the native *AtZIP1* is known to be up regulated only under conditions of zinc deficiency (Grotz et al., 1998), cassava plants expressing *AtZIP1* using a constitutive and a tuber specific promoter accumulated higher concentrations of Zn in their storage roots under Zn-sufficient conditions as compared to the non-transgenic control. The maximum zinc concentrations recorded in the storage roots of transgenic lines grown in a growth chamber was 73 mg/kg DW, which was nine times higher than non-transgenic controls. An elevated zinc concentration of 34 mg/kg DW in storage roots was also achieved when *AtMTP1* (van der Zaal et al., 1999) was expressed using the patatin promoter. Surprisingly *AtMTP1* expression using the constitutive FMV promoter did not increase tuber zinc concentrations. Transgenic plants were also produced in which the two transporters were co-expressed on the same construct (FMV-*AtZIP1* and PAT-*AtMTP1)* in an effort to further enhance the effects of each gene and to boost both uptake and storage of zinc in the vacuole. Only four transgenic lines were recovered carrying the two genes and zinc concentration in roots of these plants was not statistically different than the non-transgenic control. Results obtained when plants were grown in soil and in confined field trials also showed that certain transgenic lines had higher zinc concentrations indicating the growth chamber results were translatable to the field.

In rice, the two zinc transporters *OsZIP4* and *OsZIP5* were overexpressed under the 35S and ubiquitin promoters, respectively, resulting in increased root zinc concentration but reduced levels of zinc in the leaves (Ishimaru et al., 2007 and Lee et al., 2010). Similar results were found in the present study with increased zinc accumulation in root storage tissue being associated with a reduction in the zinc levels in leaf tissues. Transgenic plants accumulating more zinc in the storage roots showed reduced shoot growth, chlorosis in the youngest leaves and occasionally death of shoot apices which we believe was due to the reduced zinc concentrations in shoots. The symptoms we observed in the shoots of plants that accumulated high levels of zinc in tuber were characteristic of zinc deficiency symptoms found in dicots and monocots (Broadley et al., 2012) and may be due to different factors. One of these possible factors causing the phenotypes seen in cassava shoot tissues could be insufficient activity of the proteins responsible for transport and distribution of zinc within the transgenic lines over expressing genes for zinc uptake and accumulation. Possible important candidates for proteins responsible for zinc distribution would be Yellow Stripe-Like proteins, responsible for loading Zn-NA complexes and/or FRD3 proteins, which act to load citrate (FDR3) into the xylem (Waters et al., 2006; White and Broadley, 2011) and *AhHMA4* that was reported to increase leaf zinc concentrations and enhance root-to-shoot translocation (Verret et al., 2004). The other possible factor contributing to the phenotype in the transgenic plants could be insufficient production of cytosolic zinc binding partners such as nicotianamine, required for intercellular movement in the leaves (Takahashi et al., 2003).

In cassava the associated impacts on plant development due to the overexpression of AtZIP1 and AtMTP1 may be due to altered partitioning of the zinc after it is taken up from the growth medium. Experiments designed to better understand zinc partitioning in the transgenic cassava lines using radiolabelled zinc applied to leaf tissues showed a significant reduction in zinc partitioning to the lower stem at two different time points (**Table 2**). Zinc partitioning to young leaves was also reduced as much as five times 48 h after treatment to the top leaves of one transgenic line when compared to controls. This information was obtained from relatively young plants at which time the transgenic plants were phenotypically indistinguishable from the non-transgenic controls, but the observed reduction in zinc partitioning to stem tissues and young leaves may explain the poor shoot growth and zinc deficiency symptoms seen in older greenhouse and field growth plants (**Table 1**; **Figure 5**). In addition to impacts on leaf zinc levels and shoot morphology, transgenic cassava plants over accumulating this mineral showed a reduction in yield. This result is similar to reports from rice and barley (Ramesh et al., 2004; Ishimaru et al., 2007; Lee et al., 2010) where increased zinc accumulation was due to transporter overexpression.

This is the first reported attempt to increase zinc concentration in the storage root of cassava. Recently, the endosperm-specific overexpression of *MTP1* was proposed as a tool for biofortification of rice with zinc (Ricachenevsky et al., 2013). Overexpressing the *AtZIP1* and *AtMTP1* genes led to significantly increased concentration in the storage roots of cassava, but evidence indicates that overall zinc homeostasis in the transgenic lines was perturbed, resulting in reduced shoot vigor and decreased yields. Increased zinc concentration in the storage root without associated detrimental impacts could have positive impacts on human nutrition in low income populations that rely on cassava as a staple food. Future approaches could therefore focus on co-expressing either *AtMTP1* or *ATZIP1* together with a root-to-shoot translocation transporter as *AtHMA4* (Mills et al., 2003; Hussain et al., 2004) in order to increase the amount of zinc transport to leaves. More basic research is also required to fully understand the pathways and the transporters that are involved in moving zinc toward the storage root of cassava, identification of the source tissue(s) that supplies zinc to the starchy root under sufficient zinc conditions and the genes that are associated with xylem and phloem loading and uploading. Such basic knowledge will help to develop and implement more rational synthetic biology approaches to achieve elevated zinc content in the starchy roots of cassava.

### Conflict of Interest Statement

The authors declare that the research was conducted in the absence of any commercial or financial relationships that could be construed as a potential conflict of interest.

## Footnotes

1 http://www.who.int/whr/2002/chapter4/en/index3.html

2 http://www.cambia.org/daisy/bioforge_legacy/3724.html

3 http://www.who.int/nutrition/publications/WHO_WFP_UNICEFstatement.pdf
